# Eleutheroside E, An Active Component of *Eleutherococcus senticosus*, Ameliorates Insulin Resistance in Type 2 Diabetic db/db Mice

**DOI:** 10.1155/2013/934183

**Published:** 2013-04-10

**Authors:** Jiyun Ahn, Min Young Um, Hyunjung Lee, Chang Hwa Jung, Seok Hyun Heo, Tae Youl Ha

**Affiliations:** ^1^Metabolism and Nutrition Research Group, Korea Food Research Institute, 516 Baekhyun, Bundang, Seongnam, Gyeonggi 463-746, Republic of Korea; ^2^Research Division, Korea Health Supplement Institute, Seongnam 463-400, Republic of Korea

## Abstract

Eleutheroside E (EE), a principal component of *Eleutherococcus senticosus* (ES), has anti-inflammatory and protective effects in ischemia heart. However, it is unknown whether it ameliorates insulin resistance and reduces hyperglycemia in diabetes. This study investigated the effect of EE-containing ES extracts, as well as EE, on hyperglycemia and insulin resistance in db/db mice. EE increased the insulin-provoked glucose uptake in C2C12 myotubes. Moreover, EE improved TNF-**α**-induced suppression of glucose uptake in 3T3-L1 adipocytes. Five-week-old db/db mice were fed a diet consisting of ES extract or EE for 5 weeks. Both were effective in improving serum lipid profiles and significantly decreased blood glucose and serum insulin levels. ES and EE supplementation effectively attenuated HOMA-IR. Glucose tolerance and insulin tolerance tests showed that EE increased insulin sensitivity. Immunohistochemical staining indicated that ES and EE protected pancreatic alpha and beta cells from diabetic damage. In addition, ES and EE improved hepatic glucose metabolism by upregulating glycolysis and downregulating gluconeogenesis in obese type 2 diabetic mice. These data suggest that EE mediates the hyperglycemic effects of ES by regulating insulin signaling and glucose utilization. The beneficial effects of EE may provide an effective and powerful strategy to alleviate diabetes.

## 1. Introduction

Diabetes is a metabolic disorder characterized by hyperglycemia and caused by increased hepatic glucose production, abnormal glucose utilization in peripheral tissues, and inadequate insulin secretion [1]. Type 2 insulin-resistant diabetes mellitus (T2DM) accounts for 90–95% of all cases of diabetes. This heterogeneous disorder afflicts an estimated 6% of the adult population in Western societies [2]. Several approaches have been recommended to reduce hyperglycemia, including increasing pancreatic insulin release by sulfonylureas, decreasing hepatic glucose production by metformin, enhancing insulin action by thiazolidinediones, and suppressing gut glucose absorption by *α*-glucosidase. However, these treatments have limited efficacy, a high likelihood of tolerability issues, and significant mechanism-based side effects [1].

*Eleutherococcus senticosus* (Rupr. & Maxim.) is also called Harms (ES), *Acanthopanax senticosus*, Siberian ginseng, or Gasiogapi in Korea. It is a well-known tonic and sedative Chinese herb that affects various diseases with its antibacterial, antifatigue [3], antioxidant [4], and immunomodulating [5] activities. The hypoglycemic activity of ES methanol extract has also been reported [6]. However, which functional component mediates the antidiabetic effect of ES remains unknown.

It has been reported that ES has several active constituents, including lignans (sesamine, eleutheroside E), glycans (eleutherans, eleutheroside D), triterpene saponins (eleutheroside I, K, L, and M), steroid glycosides (eleutheroside A), hydroxycoumarins (isoflaxidin), phenylacrylic acid derivatives (syringin), and flavones [7].

Eleutheroside E (EE) is known to reduce physical fatigue and to enhance endurance performance [3]. In addition, it has also reported to have anti-inflammatory effects by inhibiting NF-*κ*B [8] and protecting against myocardial infarction [9]. However, to date, the effects of EE on glucose uptake and insulin resistance have not yet been studied.

In the present study, we measured the effect of EE on glucose uptake in myotubes and insulin-resistant adipocytes. We also explored the hypoglycemic effect of ES and EE in T2DM db/db mice. The insulin signaling pathway in skeletal muscle and mRNA expression of hepatic metabolism-related genes were evaluated to determine the molecular mechanisms of ES and EE-induced activity.

## 2. Materials and Methods

### 2.1. Preparation of the Plant Extract and HPLC Analyses

The ES stem was supplied by Hambackje Agricultural Association (Nonsan, Republic of Korea) and identified by Professor Yong Mok Park, College of Life Sciences, Cheongju University (Cheongju, Republic of Korea). It was ground in a mill and passed through 50-mesh sieve. ES powder (100 g) was extracted with 150 mL 50% ethanol for 5 hours at room temperature. The extracted solutions were concentrated at 50°C using a rotary vacuum evaporator (BUCHI, Flawil, Switzerland) and lyophilized. The extraction yield was 4.13% (w/w). To determine the content of active constituents in the extract, high performance liquid chromatography (HPLC) analyses were performed using a HPLC apparatus equipped with a PU-2089 plus quaternary gradient pump, auto sampler, and an ultraviolet and refractive index detector (Jasco, Tokyo, Japan). Syringin, chlorogenic acid, eleutheroside E, and isoflaxidin were purchased from Sigma-Aldrich (St. Louis, MO, USA). The standard solutions were prepared in 70% methanol. The ES extract was dissolved in HPLC-grade methanol, filtered through a 0.45 *μ*m membrane filter, and degassed in an ultrasonic bath. Separation was performed using an XTerra RP C18 column (250 × 4.6 mm, 5.0 *μ*m; Waters, Milford, MA, USA) at 35°C. The mobile phases consisted of 1% phosphoric acid (A) and 100% acetonitrile (B) at a flow rate of 1.0 mL/min. Elution was performed using the following programmed gradient elution: 0–40 min, elution with gradually increasing concentrations of solvent B (5–40%); 40–50 min, isocratic elution with 40% solvent B; and 50–60 min elution with gradually decreasing concentrations of solvent B (40–5%). Detection was performed at 216 nm.

### 2.2. Glucose Uptake Assays

C2C12 (ATCC, Manassas, VA, USA) cells were maintained in DMEM supplemented with 10% FBS, 30 *μ*g/mL penicillin, and 100 *μ*g/mL streptomycin. C2C12 myoblast differentiation was induced by switching confluent cells to DMEM supplemented with 2% horse serum and allowing formation of myotubes with daily media changes. Cells were used in experiments 4 days after differentiation. C2C12 cells were exposed to 10 *μ*M syringin, EE, or isoflaxidin for 24 h.

3T3-L1 fibroblasts (ATCC) were maintained and differentiated as described previously [10]. To induce insulin resistance, differentiated 3T3-L1 adipocytes were treated with 20 ng/mL recombinant mouse TNF-*α* (Sigma Aldrich) for 6 h. The insulin-resistant 3T3-L1 adipocytes were treated with 10 *μ*M EE for 24 h.

A fluorescent glucose analog, 2-[N-(7-nitrobenz-2-oxa-1,3-diazol-4-yl) amino]-2-deoxyglucose (2-NBDG, Invitrogen, Carlsbad, CA, USA), was used to measure glucose uptake. After exposure to the compounds described above, 500 *μ*M 2-NBDG was added to the culture media for a 10 min incubation. Cells were washed with Krebs's buffer and incubated with 100 nM insulin for 10 min. To stop the response, cells were washed with ice-cold Krebs's buffer and the fluorescence intensity of 2-NBDG was measured at an excitation wavelength of 480 nm and an emission wavelength of 540 nm.

### 2.3. Animals, Intraperitoneal Glucose Tolerance Tests (IPGTTs), and Insulin Tolerance Tests (IPITTs)

Five-week-old male db/db mice were obtained from SLC (Hamamatsu, Japan). The db/db mouse has the mutation of leptin receptor and is a model of metabolic syndrome with T2DM [11]. After acclimation for 1 week, mice were maintained for 5 weeks on AIN-76 based diet (DM) or a diet containing 0.05% or 0.1% ES extract (ESL, ESH, resp.), or 0.003% EE. The body weight and 4 h fasting blood glucose of each mouse were monitored weekly.

Five weeks after feeding, intraperitoneal glucose tolerance tests (IPGTTs) and insulin tolerance tests (IPITTs) were performed. IPGTT was determined in response to intraperitoneal administration of 2 g D-glucose/kg body weight following a 4-hour fast. Blood glucose was measured from the tail vein 0, 15, 30, 60, 90, and 120 minutes after glucose administration. IPITT was determined in response to intraperitoneal administration of 1.2 IU human insulin/kg body weight following a 4-hour fast. Blood glucose was measured 0, 15, 30, 60, 90, and 120 minutes after insulin administration. The area under the curve (AUC) was calculated using the trapezoidal method. After 5 weeks on the diet, mice were sacrificed following a 12-hour fast. All animal studies were conducted in accordance with a protocol approved by the Institutional Animal Care and Use Committee of the Korea Food Research Institute.

### 2.4. Blood Parameters

Blood glucose, triglycerides (TG), free fatty acids (FFAs), total cholesterol (TC), and high-density lipoprotein (HDL) levels were measured enzymatically using commercial kits (Shinyang Chemical Co., Busan, Republic of Korea). Serum insulin levels were measured using an ELISA kit (ALPCO Diagnostics, Salem, NH, USA). Homeostasis model assessment for insulin resistance (HOMA-IR) was calculated using the following formula:

(1) HOMA-IR(mmol/L×μU/mL)  =fasting  glucose(mmol/L)   ×fasting  insulin(μU/mL)/22.5.

### 2.5. Histological Examination

For histological analyses, pancreatic tissues were fixed in 10% buffered formalin, embedded in paraffin, sectioned, and stained with hematoxylin and eosin. The stained areas were observed using a light microscope (Olympus, Tokyo, Japan) with a magnifying power of ×200. For immunohistochemistry, pancreatic sections were deparaffinized and rehydrated, and incubated for 25 minutes in 70% methanol and hydrogen peroxide (H_2_O_2_). After washing with Tris-buffered-saline (TBS, pH 7.3), the sections were incubated overnight at 4°C with an anti-insulin antibody (BioGenex, Fremont, CA, USA) diluted 1 : 1000 in TBS containing 10% bovine serum. Sections were incubated for 90 minutes with an anti-guinea pig antibody (Vectastain, Vector, Servion, Switzerland) in TBS containing 10% bovine serum (dilution 1 : 200). After washing, sections were incubated with an avidin-peroxidase complex (Vectastain) for 15 minutes and washed again. The sections were stained with 3,3 diaminobenzidine (DAB) for 5 minutes and counter-stained with hematoxylin for 30 seconds.

Relative beta cell volume in the pancreas was described as the number of points corresponding to the anti-insulin antibody-stained area/number of points corresponding to remaining pancreatic area.

### 2.6. Insulin Signaling

For insulin signaling experiments, mice were intraperitoneally injected with 5 U/kg human insulin (Sigma Aldrich) following an overnight fast. After 5 minutes, muscle tissues were removed and frozen in liquid nitrogen. Tissues were lysed in RIPA buffer and western blot analyses were performed as previously described [10]. Primary antibodies used included phospho-AKT, phospho-P70S6 kinase (P70S6K), phospho-insulin receptor beta subunit (IR*β*), and *β*-actin (Cell Signaling, Danvers, MA, USA).

### 2.7. Quantitative Real Time Reverse Transcription-Polymerase Chain Reaction (qRT-PCR)

Livers were excised, immediately snap-frozen, and stored at −80°C until use. Total RNA was extracted from 20–30 mg of each liver tissue using a NucleoSpin RNA II kit (Macherey-Nagel GmbH & Co. KG, Duren, Germany) according to the manufacturer's protocol. RNA quantification in the extracted samples was performed using NanoDrop (Thermo Scientific). RNA purity was assessed by measuring the *A*_260_/*A*_280_ ratio. Values of 1.9–2.1 were used in experiments. Total RNA (1 *μ*g) was used for cDNA synthesis with the Maxime RT-PCR PreMix Kit (Intron, Seongnam, Republic of Korea) per the manufacturer's instructions. The total reaction volume was 20 *μ*L. qPCR was conducted on an StepOnePlus Real-time PCR system (Applied Biosystems) using SYBR Green Real-time PCR Master Mix (TOYOBO, Osaka, Japan) with a 95°C predenaturation for 5 minutes, followed by 40 cycles of 95°C for 15 seconds, 60°C for 15 seconds, and 75°C for 45 seconds. The PCR reaction was performed in a 20 *μ*L reaction volume containing 2 *μ*L cDNA template and 10 pmol primers (forward and reverse, resp.). The primers used were as follows: glucokinase, forward, 5′-AGAAGGCTCAGAAGTTGGAGAC-3′, reverse, 5′-GGATGGAATACATCTGGTGTTTCG-3′; 6-phosphofructokinase, forward, 5′-TGTGGTCCGAGTTGGTATCTT-3′, reverse, 5′-GCACCTCCAATCACTGTGCC-3′; glucose-6-phosphatase (G6Pase), forward, 5′-GAGTCTTGTCAGGCATTGCT-3′, reverse, 5′-GGTACATGCTGGAGTTGAGG-3′; phosphoenolpyruvate carboxykinase (PEPCK), forward, 5′-AAAAGCCTTTGGTCAACAAC-3′, reverse, 5′-AAACTTCATCCAGGCAATGT-3′; and 18S, forward, 5′-CTCAACACGGGAAACCTCAC-3′, reverse, 5′-CGCTCCACCAACTAAGAACG-3′.

### 2.8. Statistical Analyses

Results are expressed as the mean ± standard deviation (SD) for cell studies and the mean ± standard error (SEM) for animal studies. Statistical analyses were performed using GraphPad Prism 5 software (San Diego, CA, USA). One-way analysis of variance (ANOVA) was used to compare quantitative data among groups. The Bonferroni post hoc test was used if ANOVA indicated significance (*P* < 0.05).

## 3. Results

### 3.1. Analyses of the Functional Components of ES

**Figure 1:**
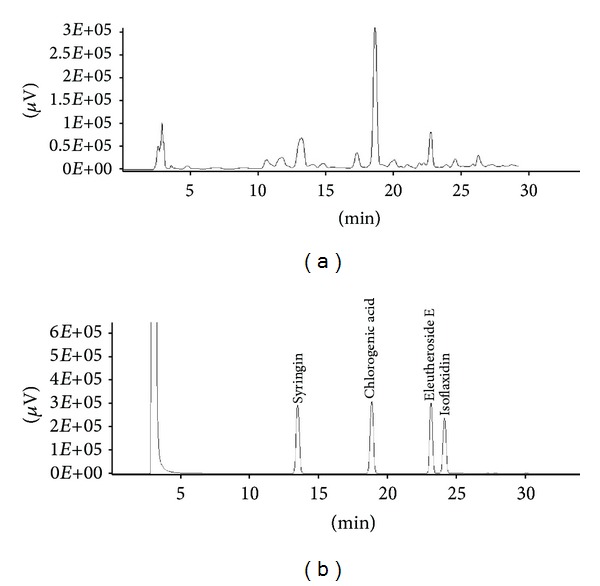
Representative HPLC chromatogram of the *E. senticosus* (ES) extract and its functional standard compounds. (a) HPLC chromatogram of the ES extract. (b) HPLC chromatogram of the major compounds including syringin, chlorogenic acid, eleutheroside E, and isoflaxidin.

The functional compounds of the 50% ethanol extract of ES were analyzed using an established HPLC method. The chromatogram of the ES extract is shown in Figure 1. The content of each active compound is presented in Table 1. The ES extract contained 16.78, 64.8, and 10.72 mg/g extract of syringin, chlorogenic acid, and EE, respectively. Previous research indicates that syringin, EE, chlorogenic acid, and isoflaxidin are the major components contributing to the pharmacological effects of ES [12]. However, isoflaxidin was not detected in our ES samples.

### 3.2. Eleutheroside E Increases Insulin-Evoked Glucose Uptake

**Figure 2:**
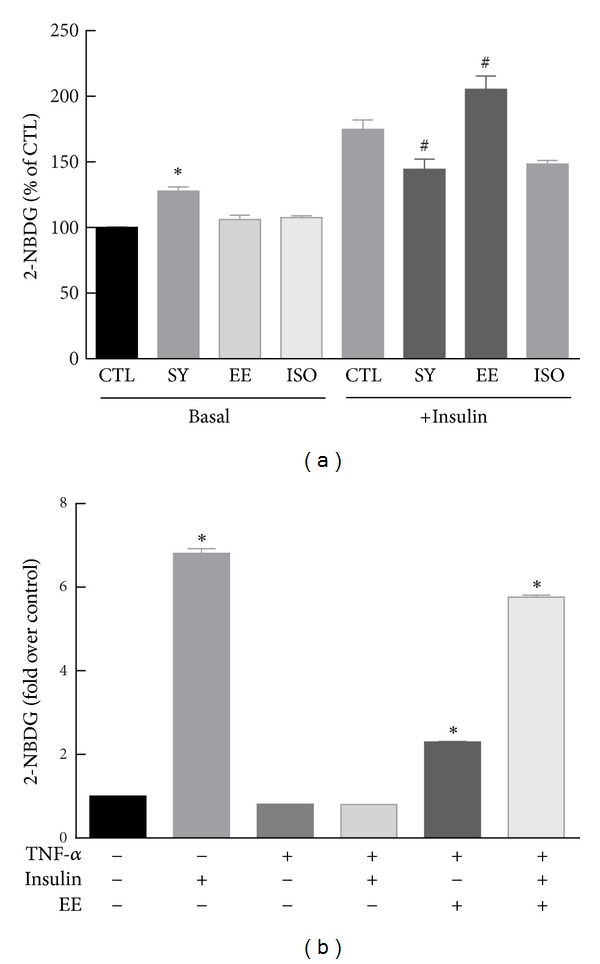
The effect of functional ES components on glucose uptake in C2C12 myotubes and insulin-resistant 3T3-L1 cells. (a) C2C12 myotubes were treated for 24 h with 10 *μ*M of each compound and glucose uptake was measured in basal and insulin exposure conditions. *P < 0.05 versus basal control. ^#^ *P* < 0.05 versus insulin-treated control. CTL, control; SY, syringin; EE, eleutheroside E; ISO, isoflaxidin. (b) For insulin resistance conditions, 20 ng/mL TNF-*α* was added for 6 h in differentiated 3T3-L1 cells. After treatment with 10 *μ*M EE for 24 h, glucose uptake was measured. *P < 0.05 versus control. Results are the mean ± SD of 6 wells in each group. Each experiment was repeated at least three times.

First, we examined the effects of the functional components of ES on glucose uptake in muscle myotubes. As shown in Figure 2(a), syringin increased basal glucose uptake in C2C12 myotubes. EE noticeably amplified the insulin-stimulated glucose uptake.

To evaluate the effect of EE on impaired glucose uptake in an insulin-resistant state, we induced insulin resistance using TNF-*α* treatment in 3T3-L1 adipocytes and measured the effect of EE on TNF-*α*-mediated suppression of glucose uptake. Treatment of 10 *μ*M EE for 24 h increased basal glucose uptake as well as improved TNF-*α*-mediated suppression of glucose uptake (Figure 2(b)). These data suggest that EE increases glucose uptake and could have a beneficial role in insulin-resistant diabetes.

### 3.3. Diet Containing Eleutheroside E Ameliorates Diabetes in db/db Mice

**Figure 3:**
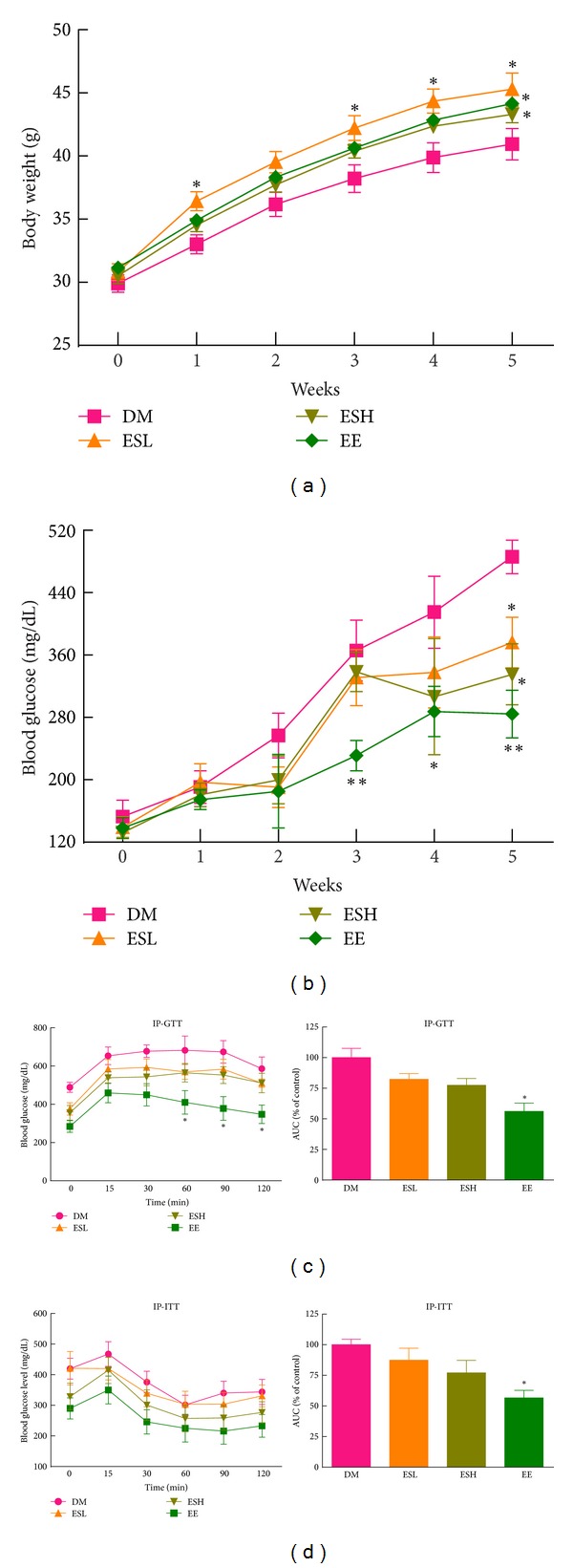
The effects of ESL, ESH, and EE on body weight, fasting blood glucose levels, and impaired glucose tolerance in db/db mice. (a) Body weight measurements during the experimental period. (b) Blood glucose levels after 4-hour fast. After feeding the experimental diet for 5 weeks, IPGTT (c) and IPITT (d) were performed. The area under curve (AUC) during IPGTT is also shown. Each value represents the mean ± SEM (*n* = 8). *P < 0.05 versus diabetes mellitus (DM) group. **P < 0.01 versus DM group.

To confirm the effect of EE on hyperglycemia and glucose intolerance, we supplemented db/db mice with an experimental diet containing ESL, ESH, or EE for 5 weeks. Changes in the body weights of animals during the experimental period are shown in Figure 3(a). There was a progressive increase in body weight for 5 weeks, and the final body weights of animals in the experimental groups (ESL, ESH, and EE) were significantly higher than control animals.

As shown in Table 2, serum TC and TG levels were significantly decreased after ESL and ESH treatment. EE effectively lowered TG levels. Serum FFA levels were significantly reduced by ESL and EE treatment.

The diabetic control group showed higher fasting blood glucose and insulin levels and ESL, ESH, and EE treatments effectively decreased fasting blood glucose and insulin levels. Moreover, ESL, ESH, and EE treatment significantly reduced HOMA-IR values compared to the diabetic control group.

To evaluate the effect of ES and EE on the insulin resistance of db/db mice, we performed IPGTT and IPITT at 5 weeks of treatment. After intraperitoneal glucose injection, blood glucose levels were monitored. As shown in Figure 3(c), both ES and EE improved impaired glucose tolerance, although ES treatment did not reach statistical significance. The area under curve (AUC) decreased 17.7%, 22.6%, and 43.9% in the ESL, ESH, and EE groups, respectively, compared with the diabetic control group.

For IPITT (Figure 3(d)), ES and EE treatment modestly ameliorated the impaired insulin action compared with the diabetic control group. The AUC of IPITT was significantly decreased in EE-treated mice compared to the diabetic control mice. Collectively, these results indicate that ES and EE have hypoglycemic effects and improve glucose tolerance.

**Figure 4:**
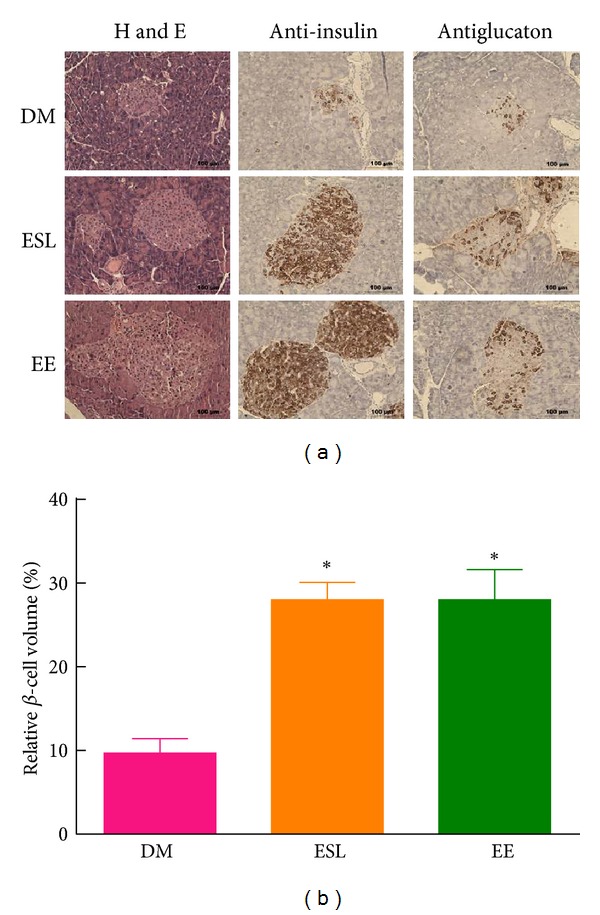
The effect of ESL and EE on pancreatic insulin and glucagon production. (a) Pancreatic tissue sections from DM, ESL, and EE group were stained with hematoxylin and eosin, anti-insulin, and antiglucagon antibodies, respectively. The scale bar is 100 *μ*m for each panel. (b) Relative *β*-cell volume in the pancreas was calculated as described in Materials and Methods. *P < 0.05 versus diabetes mellitus (DM) group.

We hypothesized that the improvement of glucose tolerance by ES and EE resulted from protection of *β*-cell function. To test this hypothesis, we examined the effect of ES and EE on the function of pancreatic *α*- and *β*-cells (Figure 4(a)). The insulin content of *β*-cells was determined using immunochemistry and we found that ESL and EE effectively prevented diabetic loss of *β*-cells (Figure 4(b)). Furthermore, ESL and EE ameliorated impairment of *α*-cells as confirmed by immunohistological glucagon staining (Figure 4(a)). These results suggest that supplementation of ES and EE effectively prevent diabetic impairment of pancreatic *α*- and *β*-cells.

**Figure 5:**
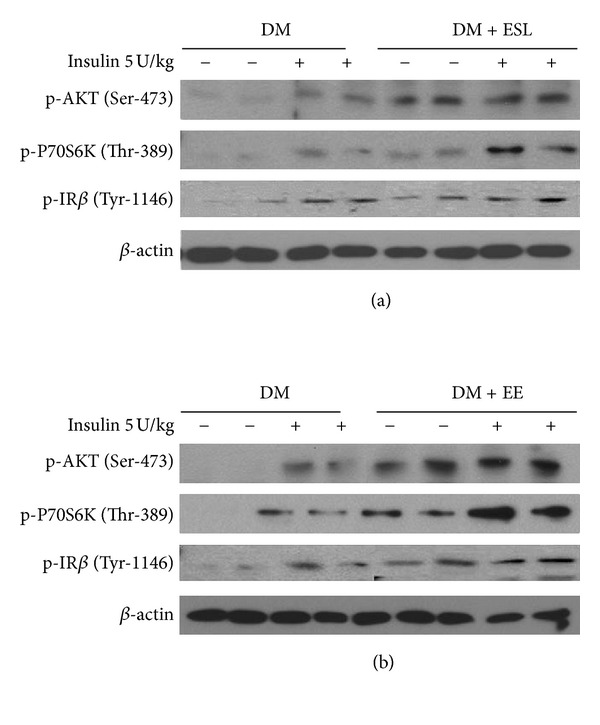
The effect of ESL (a) and EE (b) on insulin signaling in skeletal muscles. Analyses of insulin-induced phosphorylation of IR*β*, AKT, and P70S6K were performed by western blot. Following an overnight fast, mice were either sacrificed for or injected with 5 U/kg of insulin. Five minutes after injection, muscle tissues were collected and total protein was analyzed. The phosphorylation site in each protein is indicated.

To further explore the effects of ES and EE on insulin sensitivity, insulin signaling was evaluated after insulin injection in muscle of overnight-fasted mice from the ESL- and EE-treated groups. As shown in Figure 5(a), IR*β* phosphorylation was decreased in diabetic mice. Phosphorylation of AKT and its downstream target, P70S6K, was also reduced. However, ESL supplementation maintained insulin-induced phosphorylation of IR*β*, AKT, and P70S6K. Similar to ESL, EE treatment increased insulin-dependent phosphorylation of IR*β*, AKT, and P70S6K compared to diabetic mice (Figure 5(b)).

**Figure 6:**
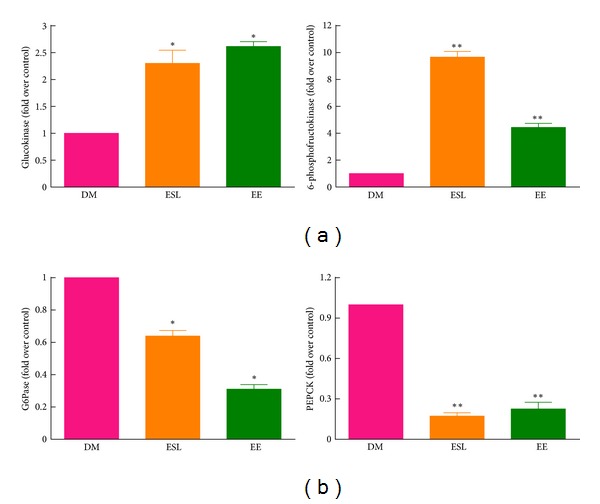
The effect of ESL and EE on hepatic glucose metabolism. (a) mRNA expression of the glycolysis-related genes, glucokinase and 6-phosphofructokinase, is shown. (b) mRNA expression of gluconeogenesis-related genes, G6Pase and PEPCK, is shown. After normalization of each gene to the 18S gene, mRNA levels are expressed as the percentage of diabetic mice. Values indicate the mean ± SEM. *P < 0.05 versus DM group. **P < 0.01 versus DM group.

Finally, we examined the effect of ES and EE on hepatic glucose metabolism by measuring the expression of genes involved in glycolysis and gluconeogenesis. As shown in Figure 6(a), ESL and EE supplementation significantly increased the mRNA expression of glucokinase and 6-phosphofructokinase. In contrast, ESL and EE markedly decreased the mRNA expression of G6Pase and PEPCK (Figure 6(b)). These data indicate that ES and EE improved glucose metabolism by upregulating glycolysis and downregulating gluconeogenesis in diabetic mice.

## 4. Discussion

T2DM is a common metabolic disorder characterized by chronic hyperglycemia and dyslipidemia resulting from peripheral tissue insulin resistance and impaired insulin secretion from the pancreas [13]. It is estimated that T2DM patients lose 15 years of an average life expectancy [14]. Therefore, intensive treatment to control hyperglycemia is needed in diabetes. However, because clinical therapies have limited efficacy and significant mechanism-based side effects, there have been enormous increases in the use of medicinal plants for diabetes management [15].

In the present study, we have shown that treating diabetic db/db mice with ES and its main component, EE, improves glycemic control and insulin resistance. We also report that ES and EE have protective effects on pancreatic *β*-cell function. This study provides the first evidence that the EE-induced positive effects are mediated by the enhancement of insulin signaling and the recovery of dysregulated glucose metabolism.

ES has been reported to contain syringin, eleutheroside E, isoflaxidin, and chlorogenic acid as the main compounds [12]. In accordance with previous studies, our ES extract was found to contain syringin, chlorogenic acid, and eleutheroside E. However, isoflaxidin was not detected in our sample.

The hypoglycemic activity of the ES extracts was demonstrated and syringin [6] and chlorogenic acid [16] were suggested to be the bioactive compounds. However, little attention has been given to the role of EE on hyperglycemia and glucose utilization. Thus, we focused on how EE affects glucose uptake and insulin resistance.

Impaired glucose uptake to peripheral tissues results in high circulating glucose levels [17]. Therefore, the main strategy is to stimulate glucose uptake to skeletal muscle, liver, and adipocytes that consume most plasma glucose. EE increased insulin-evoked glucose uptake in C2C12 myotubes. Moreover, EE recovered impaired glucose uptake in TNF-*α* induced insulin-resistant adipocytes. These results suggest that EE has the potential to alleviate insulin resistance.

To investigate this hypothesis further, we examined the effect of dietary EE on hyperglycemia and glucose metabolism using db/db mice. We found that ES, as well as EE, inhibited hyperglycemia and glucose tolerance. In addition, hypertriglyceridemia and increased FFAs were improved upon ES and EE treatment. Plasma FFAs are derived almost exclusively from adipose tissue through lipolysis of TG. It has been demonstrated that increased plasma FFA concentration causes insulin resistance and is associated with T2DM [18]. These findings led us to conclude that EE is one of the functional ES components for the attenuation of diabetes.

A key component of the pathophysiology of T2DM involves defective insulin secretion from pancreatic islet *β*-cells. This defect leads to the failure of the *β*-cell to compensate for insulin resistance and successive hyperglycemia [19]. Our animal model, db/db mice, is characterized by reduced proliferation and increased apoptosis in pancreatic *β*-cells [20]. The present study reveals the pharmacological effects of EE in pancreatic islet function and pathological development. Compared with diabetic mice, higher numbers of insulin secreting *β*-cells and glucagon producing *α*-cells, as well as functional integrity islet morphology, were observed in ES- or EE-treated mice. The protective effect of ES and EE on *β*-cell destruction and the subsequent enhancement of *β*-cell function improved glycemic control.

Insulin resistance is an important risk factor in the development of T2DM. It results from multiple defects in signal transduction pathways, including reduced tyrosine phosphorylation of IR and subsequently decreased phosphorylation of AKT and its direct target, P70S6K [21]. Increasing the phosphorylation of these molecules enhances insulin-mediated signal transduction. We performed western blot analyses and confirmed that ES and EE treatment recovered impaired insulin signaling in skeletal muscle tissues by increasing the insulin-induced phosphorylation of IR*β*, AKT, and P70S6K.

The liver has a critical role in regulating endogenous glucose production from *de novo* synthesis (gluconeogenesis) or glycogen catabolism (glycogenolysis). Increased hepatic glucose production is predominantly responsible for the development of hyperglycemia in diabetes [22]. Thus, reducing excessive hepatic glucose production is a good strategy to alleviate diabetes [23]. Several enzymes that regulate rate-controlling steps in gluconeogenic or glycolytic pathways are obvious molecular targets for therapeutic treatment. In our study, ES and EE were shown to upregulate glucokinase and 6-phosphofructokinase and downregulate G6Pase and PEPCK, thereby upregulating glycolysis and downregulating gluconeogenesis, respectively.

In conclusion, the present study provides evidence that EE ameliorates type 2 diabetes by enhancing glucose uptake, improving insulin resistance and pancreatic islet cell function, and regulating glucose metabolism. These results suggest that EE may be partially responsible for the antidiabetic effects of ES.

## Figures and Tables

**Table 1:** The functional constituents in *E. senticosus* extracts.

(mg/g extract, w/w)	Syringing	Chlorogenic acid	Eleutheroside E
	16.78 ± 0.18	64.80 ± 0.79	10.72 ± 0.19

Data are expressed as the mean ± SD from at least three measurements.

**Table 2:** The effect of ES and EE on the lipid profile of db/db mice.

	DM	ESL	ESH	EE
Total cholesterol (mg/dL)	375.11 ± 74.79	238.19 ± 86.42*	247.29 ± 45.91*	330.59 ± 21.17
Triglyceride (mg/dL)	180.24 ± 57.19	137.89 ± 35.77	113.93 ± 20.17*	112.35 ± 29.75*
FFA (uEq/L)	1412.78 ± 74.28	1080.16 ± 160.21*	1190.08 ± 102.30	1072.38 ± 269.91*
Glucose (mg/dL)	486 ± 21.53	397.8 ± 29.30*	389.2 ± 21.24*	315.0 ± 24.67*
Insulin (ng/mL)	8 ± 1.8	2.81 ± 0.71*	4.28 ± 1.29*	3.56 ± 0.41*
HOMA-IR	75.89 ± 13.64	36.49 ± 12.22*	42.79 ± 15.95*	33.5 ± 6.63*

All values are expressed as the mean ± SEM. Six-week-old db/db mice were fed experimental diet containing none, 0.05–0.01 % *E. senticosus* (ES) extract, and 0.1% eleutheroside E (EE) for 5 weeks.

DM: diabetes mellitus group; ESL: 0.05% ES extract supplemented group; ESH: 0.1% ES extract supplemented group; EE: 0.003% eleutheroside E supplemented group.

*P < 0.05 when compared to the DM group using one-way ANOVA followed by post hoc Bonferroni's tests.

## Abbreviations

ES: *Eleutherococcus senticosus*
EE: Eleutheroside E
T2DM: Type 2 insulin-resistant diabetes mellitus
HPLC: High performance liquid chromatography
2-NBDG: 2-[N-(7-nitrobenz-2-oxa-1,3-diazol-4-yl) amino]-2-deoxyglucose
IPGTTs: Intraperitoneal glucose tolerance tests
IPITTs: Intraperitoneal insulin tolerance tests
AUC: Area under the curve
TG: Triglycerides
FFAs: Free fatty acids
TC: Total cholesterol
HDL: High-density lipoprotein
HOMA-IR: Homeostasis model assessment for insulin resistance
P70S6K: P70S6 kinase
IR*β*: Insulin receptor beta subunit
G6Pase: Glucose-6-phosphatase
PEPCK: Phosphoenolpyruvate carboxykinase
ANOVA: One-way analysis of variance.
